# Magnetic resonance imaging characteristics of intramuscular lipomas: Características de lipomas intramusculares em imagem de ressonância magnética

**DOI:** 10.1590/1516-3180.2014.86200716

**Published:** 2014-11-07

**Authors:** Ivan Chernev, Nadege Petit-Clair

**Affiliations:** I MD. Clinical Assistant Professor of Physical Medicine and Rehabilitation, West Virginia School of Osteopathic Medicine, Lewisburg, WV, USA. Attending Physician in Physical Medicine and Rehabilitation, Department of Medicine, Appalachian Regional Healthcare, Beckley, WV, USA.; II Medical Student, Avalon University School of Medicine, Youngstown, OH, USA.

Dear Editor,

Intramuscular lipoma is a deep-seated soft tissue lipoma that arises in muscle tissue, and is benign and relatively rare. Although multiple reports exist regarding intramuscular lipomas, their clinical and imaging characteristics are still not very well-known by clinicians. In general, we believe that there are three subtypes of intramuscular lipomas, which can define some of their imaging differences. These subtypes are infiltrative, well-defined/encapsulated and mixed, or partially well-defined/partially encapsulated (with areas of infiltration and well-defined areas).^1^ In the well-circumscribed subtype, the fatty tissue is clearly delineated from the surrounding muscle tissue, whereas in the infiltrative subtype the fatty tissue infiltrates the muscle fibers. The magnetic resonance imaging (MRI) findings do not always correspond to the histological findings in the infiltrative subtype, as some intramuscular lipomas may be minimally infiltrative at the margins and this can only be seen microscopically. When investigated, intramuscular lipomas have been commonly categorized in the same group as other deep-seated lipomas such as intermuscular lipomas or even superficial lipomas. Specifically, the MRI characteristics of different subtypes of lipomas may differ, which may create confusion among clinicians.

Recently, we read the very interesting article by Balabram et al. named “Intramuscular lipoma of the subscapularis muscle” published in the *Sao Paulo Medical Journal*.^2^ In that article, the authors briefly discussed some of the MRI characteristics of intramuscular lipomas, referencing several articles. We would like to point out that none of the referenced studies investigated the specific MRI characteristics of intramuscular lipomas independently. In fact, some of them completely excluded intramuscular lipomas. There are relatively few studies that exclusively distinguish the MRI characteristics of intramuscular lipomas.^3^^,^^4^ In addition, we are not aware of any studies directly comparing the MRI characteristics of intramuscular lipomas and deep well-differentiated liposarcomas, i.e. their main differential diagnosis. Knowing these characteristics may help in the diagnosis and in planning the surgical procedure. In this letter to the editor, we would like to summarize the MRI characteristics of intramuscular lipomas from the available literature.

On MRI, intramuscular lipomas are round or fusiform with occasional dumbbell-shaped masses. In contrast, well-differentiated liposarcomas have oblong or oval shape, with the dumbbell shape relatively frequent and crescent-shaped and fusiform masses less common. Intramuscular lipomas may vary between less than 3 cm and more than 15 cm. In general, well-differentiated liposarcomas are larger but the size alone is not significant enough to differentiate between them.^3^ Furthermore, intramuscular lipomas tend to be larger than the conventional subcutaneous lipomas.

Although some intramuscular lipomas (well-defined and marginal infiltration lipomas) may be composed of homogenously pure fatty tissue similar to superficial lipomas, heterogeneity due to intermingled fat and muscle fibers is very common and is not a sign of malignancy. Interdigitation with skeletal muscle, creating a very typical striated appearance, is characteristic of intramuscular lipomas. This feature has not been described in well-differentiated liposarcomas and allows confident diagnosis of intramuscular lipoma. Well-differentiated liposarcomas are also heterogeneous. While tumor tissue other than muscle fibers exists in liposarcomas, the tumor tissue can easily be distinguished from muscle fibers, since the latter maintain their original structure and are usually isointense with normal muscle on both T1- and T2-weighted images. Nodular and patchy signal intensities different from fat (non-adipose components) have been commonly noted in well-differentiated liposarcomas. Entrapped muscle fibers (intralesional muscle fibers) have been reported in some cases of well-differentiated liposarcomas. However, this matter is still controversial and requires more research in order to define the significance of this finding on MRI.^4^^,^^5^^,^^6^

Intramuscular lipomas may present with well-defined or irregular margins, or both. Completely irregular margins are a feature of intramuscular lipoma rather than well-differentiated liposarcomas.^7^ A capsule is rarely recorded in intramuscular lipomas and sometimes it may only be at the outer margin and not at the margin in the muscle. Furthermore, well-differentiated liposarcomas are commonly well-defined and capsulated and tend to grow expansively rather than infiltratively.

Most intramuscular lipomas are unilobular, however bilobular lipomas have been described as well.^3^^,^^8^ In comparison, the majority of well-differentiated liposarcomas are multilobular.

Variable thicknesses of streaky structures (septa) have been observed inside the lesions and between the lobes (if bilobular) in the majority of patients with intramuscular lipomas. Thick linear structures within the nodules, which have been recognized not only in well-differentiated liposarcomas but also in intramuscular lipoma, may not be pathognomonic.^3^ However, we believe that thicker linear structures are more common in well-differentiated liposarcomas.

Mild enhancement of the mass and streaky structures in intramuscular lipomas after contrast material administration can be observed occasionally. This is in contrast to well-differentiated liposarcomas, where the septa and globular areas of the tumor tend to be enhanced more prominently.

In conclusion, a single MRI characteristic of intramuscular lipomas may not be sufficient for the diagnosis. However, considering all MRI features together and careful examination of the images may often afford almost pathognomonic evidence and help in selecting appropriate surgery. Future studies should directly compare MRI features of intramuscular lipomas with deep-seated well-differentiated liposarcomas, with additional histological and cytogenetic correlations for better accuracy.
