# Supplementary material for: Magnetic resonance imaging characteristics of intramuscular lipomas: Características de lipomas intramusculares em imagem de ressonância magnética
Source: Sao Paulo Med J. 2014 Nov 7;133(1):64–6. doi: 10.1590/1516-3180.2014.86200716 (PMC10496612; doi:10.1590/1516-3180.2014.86200716)
Supplement: Related texts

# Reply: RESPONSE TO LETTER TO THE EDITOR

**DOI:** 10.1590/1516-3180.2014.86200716

**Authors:** Débora Balabram, Carla Cristina de Sousa Resende Cabral, Omar de Paula Ricardo, Cristóvão Pinheiro de Barros

**Affiliations:** III MD, PhD. Breast Surgeon, Instituto da Previdência dos Servidores do Estado de Minas Gerais (IPSEMG), Belo Horizonte, Minas Gerais, Brazil.; IV MD. Radiologist, Serviço de Radiologia e Ultrassonografia de Minas Gerais (Sermig), Belo Horizonte, Minas Gerais, Brazil.; V MD. Pathologist, Laboratory of Anatomical and Diagnostic Pathology, Belo Horizonte, Minas Gerais, Brazil.; VI MD. Breast Surgeon, Instituto da Previdência dos Servidores do Estado de Minas Gerais (IPSEMG), Belo Horizonte, Minas Gerais, Brazil.

We thank you for further describing the specific characteristics of deep-seated intramuscular lipomas and citing references not present in our article,^1^^,^^2^ entitled “Intramuscular lipoma of the subscapularis mucle”.^3^

However, we would like to point out that the main goal of our case report was to describe the unusual presentation of a deep-seated lipoma as an axillary mass. Since the patient was 58-year-old at the time of presentation, it was important to rule out metastatic breast cancer as well as other malignant diseases.^4^^,^^5^ Using the search strategy for clinical cases described in our article,^3^ we did not find similar clinical presentations of intramuscular lipomas in the literature (PubMed, Lilacs and Embase). We also did not come across descriptions of any lipomas inside the subscapularis muscle.

## References

[1] Matsumoto K, Hukuda S, Ishizawa M, Chano T, Okabe H (1999). MRI findings in intramuscular lipomas. Skeletal Radiol.

[2] Nishida J, Morita T, Ogose A (2007). Imaging characteristics of deep-seated lipomatous tumors: intramuscular lipoma, intermuscular lipoma, and lipoma-like liposarcoma. J Orthop Sci.

[3] Balabram D, Cabral CC, Filho O de P, de Barros CP (2014). Intramuscular lipoma of the subscapularis muscle. Sao Paulo Med J.

[4] de Andrade JM, Marana HR, Sarmento JM (1996). Differential diagnosis of axillary masses. Tumori.

[5] Park YM, Park JS, Yoon HK, Yang WT (2013). Imaging-pathologic correlation of diseases in the axilla. AJR Am J Roentgenol.
